# Double Pyramidal Lobe of the Thyroid Gland

**DOI:** 10.4274/balkanmedj.2017.1581

**Published:** 2018-07-24

**Authors:** Emin Gürleyik

**Affiliations:** 1 Department of Surgery, Düzce University School of Medicine, Düzce, Turkey

The pyramidal lobe is a thyroid tissue of embryologic origin. It is situated in the pretracheal region between the isthmus and the hyoid bone. The high incidence of 65.7% of patients undergoing thyroidectomy suggests that pyramidal lobe is a common component of the thyroid gland rather than an uncommon anatomic variation (1). Double pyramidal lobes that are extremely rare anatomic variations are shown in the clinical image.

A 58-year-old woman presented to our clinic with signs and symptoms of hyperthyroidism. Biochemical analysis confirmed thyrotoxicosis. Ultrasonographic examination and thyroid nuclear scan revealed solid, hot, autonomous, hyperactive multiple nodules in both lobes. The diagnosis was toxic multinodular goiter. Before the surgery, written informed consent was obtained from the patient for both surgical management and scientific publication. The patient underwent total thyroidectomy. Full dissection of the lateral lobes was performed, and both the lateral lobes were mobilized medially after completing the surgical dissection. The anterior cervical region between the isthmus and the hyoid bone was completely dissected for identifying the presence of any thyroid tissue. Two different pyramidal lobes, rare anatomic variations that originated from the junction points of the isthmus with the right and the left lobes of the gland, were observed. Both the pyramidal lobes were completely dissected from the isthmus up to the hyoid bone. The thyroid gland, including the two pyramidal lobes, was completely excised to achieve total thyroidectomy. Pathological examination of the thyroidectomy specimen revealed two large pyramidal lobes (Figure 1). Written informed consent was obtained from the patient.

Completeness of thyroidectomy has great relevance for both autoimmune and malignant diseases. Remnant tissue after surgical operation may complicate the proper treatment of such diseases and the sensitive postoperative follow-up of patients. Based on its high incidence, the pyramidal lobe is considered as a normal component of the thyroid gland that may be affected by the diseases that affect the rest of the thyroid parenchyma and uncommonly harbor malignant disease (2). Anatomic variations of the gland, remnant pyramidal lobes, preclude complete removal of thyroid tissue, which can cause recurrent goiter. 

The incidence of pyramidal lobe is significantly higher in thyroidectomy cases. In fact, surgeons generally find a single pyramidal lobe. Conversely, the presence of double pyramidal lobes is extremely rare, and we could find only three previous cases in the English literature (3,4,5). All the reported patients with double pyramidal lobes were women who had various thyroid diseases indicating surgical intervention (Table 1). If pyramidal lobe is not excised during total thyroidectomy, postoperative hypertrophy of this remnant tissue may result in recurrent disease or appear as a midline lump years after the primary operation.

The presence of pyramidal lobe is a typical example of an anatomic variation of the thyroid. The presence of two pyramidal lobes is an extremely rare occurrence that may affect the completeness of thyroidectomy. Various locations of the base of pyramidal lobe generally require careful dissection of both the pretracheal and the prelaryngeal regions from the upper border of the isthmus up to the upper border of the thyroid cartilage in most of the patients and sometimes up to the hyoid bone. Therefore, the anterior cervical region has to be dissected carefully during surgery so that no residual thyroid tissue remains.

## Figures and Tables

**Table 1:**
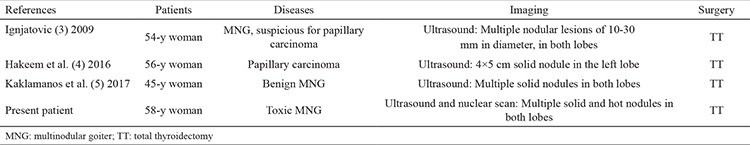
Characteristics of patients with double pyramidal lobes published in the literature

**Figure 1:**
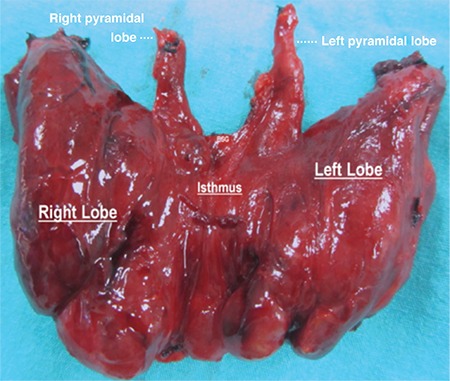
Two pyramidal lobes arising from the junction points of the isthmus with both lateral lobes.

## References

[1] Gurleyik E, Gurleyik G, Dogan S, Cobek U, Cetin F, Onsal U (2015). Pyramidal lobe of the thyroid gland: surgical anatomy in patients undergoing total thyroidectomy. Anat Res Int.

[2] Yoon SG, Yi JW, Seong CY, Kim JK, Kim SJ, Chai YJ, et al (2017). Clinical characteristics of papillary thyroid carcinoma arising from the pyramidal lobe. Ann Surg Treat Res.

[3] Ignjatovic M (2009). Double pyramidal thyroid lobe. J Postgrade Med.

[4] Hakeem AH, Hakeem IH, Wani FJ (2016). Double pyramidal lobe of thyroid gland: A rare presentation. Thyroid Research Practice.

[5] Kaklamanos I, Zarokosta M, Flessas I, Zoulamoglou M, Katsoulas T, Birbas K, et al (2017). Surgical anatomy of double pyramidal lobe on total thyroidectomy: a rare case report. J Surg Case Rep.

